# Cross-species single-cell transcriptomic analysis reveals divergence of cell composition and functions in mammalian ileum epithelium

**DOI:** 10.1186/s13619-022-00118-7

**Published:** 2022-05-05

**Authors:** Haonan Li, Xiaodan Wang, Yalong Wang, Mengxian Zhang, Fan Hong, Hong Wang, Along Cui, Jianguo Zhao, Weizhi Ji, Ye-Guang Chen

**Affiliations:** 1 The State Key Laboratory of Membrane Biology, Tsinghua-Peking Center for Life Sciences, School of Life Sciences, Tsinghua University, Beijing, 100084 China; 2 Guangzhou Institutes of Biomedicine and Health, Chinese Academy of Sciences, Guangzhou Science Park, Guangzhou, 510530 China; 3 Guangzhou Laboratory, Guangzhou, 510005 China; 4 State Key Laboratory of Primate Biomedical Research, Institute of Primate Translational Medicine, Kunming University of Science and Technology, Kunming, 650500 Yunnan China; 5 State Key Laboratory of Stem cell and Reproductive Biology, Institute of Zoology, Chinese Academy of Sciences, Beijing, 100101 China

**Keywords:** scRNA-seq, Transcriptome, Ileum, Monkey, Pig, Rat, CA7 cells

## Abstract

Animal models are widely used for biomedical studies and drug evaluation. The small intestine plays key roles in nutrient absorption, hormone secretion, microbiota defense and drug absorption and metabolism. Although the intestinal structure of mammals is conserved, the differences on epithelial cell composition, functional assignments and drug absorption among mammals are largely unknown. Here, cross-species analysis of single-cell transcriptomic atlas of the ileum epithelium from mouse, rat, pig, macaque and human reveals the conserved and differential cell types and functions among species, identifies a new CA7^+^ cell type in pig, macaque and human ileum, uncovers the distinct expression pattern in enterocytes, enteroendocrine cells and Paneth cells, and defines the conserved and species-specific intestinal stem cell signature genes. The examination of drug absorption across species suggests that drug metabolism in mouse ileum is closer to human while drug transport in macaque ileum is more similar to human. Together, our data provide the comprehensive information about cell composition and functional assignments in five species, and offer the valuable guidance for animal model selection and drug testing.

**Supplementary Information:**

The online version contains supplementary material available at 10.1186/s13619-022-00118-7.

## Background

Animal models have been widely used for the understanding of human physiological and pathological processes and for preclinical drug evaluation. Although it is well recognized that the drug efficacy and toxicity response in animal models could be finely extrapolated to humans, there are limitations due to interspecies differences (Lin, 1995; Martignoni et al., 2006). The interspecies differences are attributed to anatomic morphology, digestive function and the gene expressions involved in drug metabolism and transport (Kararli, 1995; Martinez et al., 2002; Xu et al., 2021). For example, pigs are not a good choice to evaluate drug candidates involved in sulfate conjugation, including paracetamol and tamoxifen (Dalgaard, 2015; Marto et al., 2017). Therefore, a comprehensive understanding of anatomic morphology and drug absorption across species is pivotal for animal model selection of drug development. Single-cell mRNA sequencing (scRNA-seq) provides a transcriptomic landscape at the single-cell level and has broadened the understanding of the cell composition of numerous organs (Han et al., 2018; Quake, 2022). Cross-species single-cell transcriptome comparison also reveals the conserved and divergent features of cell types, signaling patterns and development process in several organs including brain (Franjic et al., 2021; Geirsdottir et al., 2020; Hodge et al., 2019), lung (Raredon et al., 2019) and testis (Lau et al., 2020).

The small intestine is responsible for nutrient absorption (Zorn and Wells, 2009), hormone secretion (Sanger and Lee, 2008) and resistance to microbial invasion (Peterson and Artis, 2014). The structure of small intestine in mammals is highly conserved and comprises the crypt and villus region (Furness et al., 2015; Kararli, 1995; Lickwar et al., 2017). Despite the comprehensive study about cell types of intestinal epithelium in mouse and human (Haber et al., 2017; Wang et al., 2020b), cross-species analysis of the intestine in the single-cell level is less understood. Here, we provided the cross-species single-cell transcriptomic atlas of mouse, rat, pig and cynomolgus monkey ileum. With the published scRNA-seq data from human ileum (Wang et al., 2020b), we analyzed the conserved and differential cell types and functions among species, identified a new CA7^+^ cell subtype in pig, macaque and human ileum, and defined the conserved and species-specific intestinal stem cell signature genes. Furthermore, our analyses uncover the difference on drug absorption across species. These data provide the valuable guidance for animal model selection in disease dissection and drug development.

## Results

### Generation of cross-species single-cell transcriptomic atlases of ileal epithelium

**Fig. 1:**
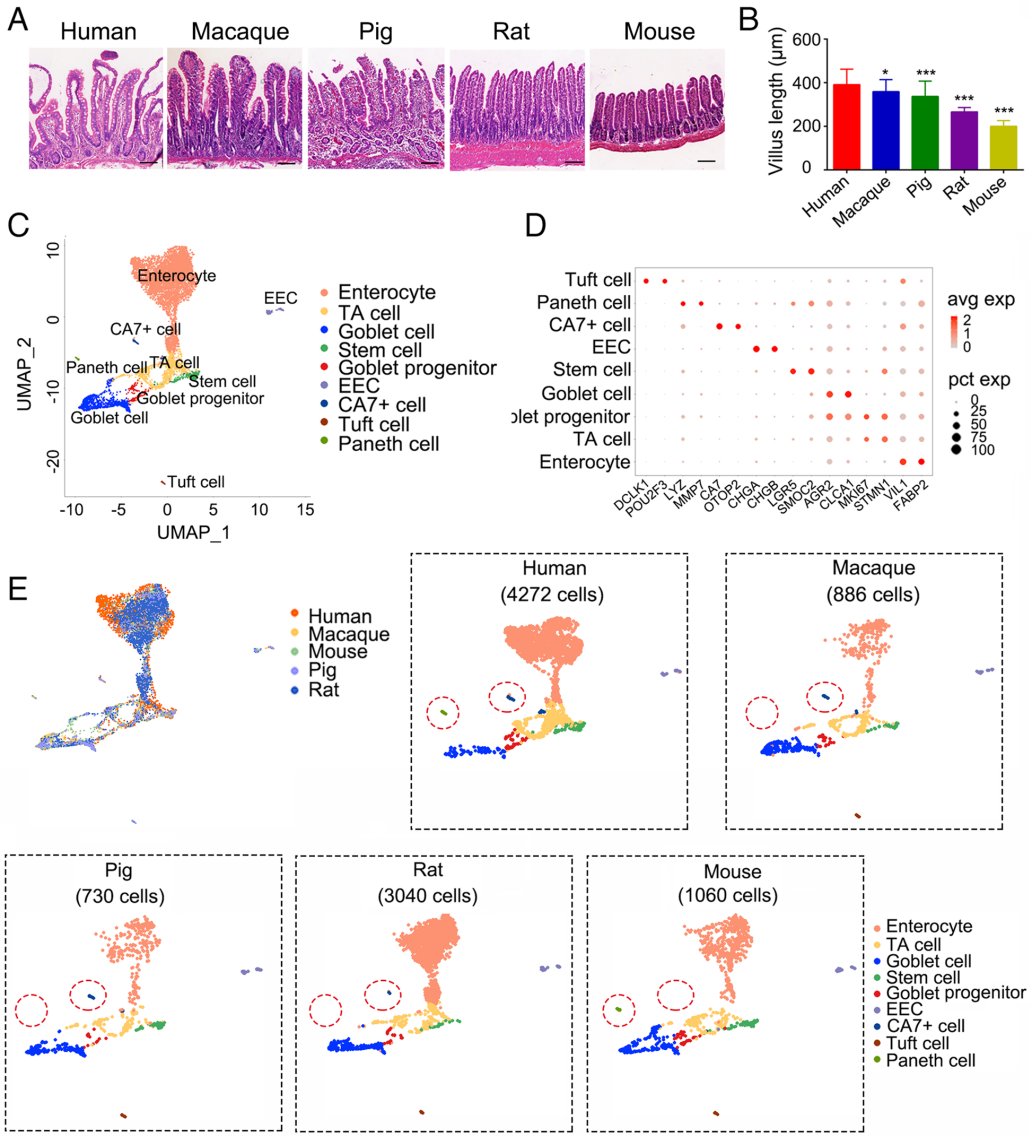
Generation of the transcriptional atlases of cross-species ileum epithelial cells. **A**, **B,** H&E staining on ileum sections (**A**) and quantitation of the length of villus (**B**) from human, macaque, pig, rat and mouse. Scale bars, 100 μm. Data are displayed as the mean ± SD by one-way ANOVA. 50 villus regions were calculated for the length in **B**. The significance was displayed by each species compared to human. *P < 0.05, **P < 0.01, ***P < 0.001. **C,** UMAP plots showing different cell types from 9988 ileum epithelial cells from human, macaque, pig, rat and mouse. **D,** Dot Plot showing cell type-specific genes, colored by relative gene expression. Each dot represents a gene and the size shows the percentage of cells expressing this gene. **E,** UMAP plot of ileum epithelial cells grouped by species. The red circles point differential cell types across species, including Paneth cells and CA7^+^ cells, respectively

To determine intestinal conservation across species, we collected ileum tissues from mice, rats, pigs, macaques and humans. The overall morphology of the ileum was similar, comprising the crypt and villus region across species (Fig. 1A). Human villi were the longest, followed by macaque (Fig. 1B).

Then, we performed scRNA-seq analysis to characterize the cell landscapes of the ileal epithelium from mice, rats, pigs and macaques with 10x genomics system, and scRNA-seq of human ileum was downloaded from the published data (Wang et al., 2020b). After quality filtering, the transcriptome profiles of total 9988 cells were obtained, which yielded an average of 2673 detected genes per cell (Table S1). To compare the ileum from each species, we collected 10,118 “1–1–1-1-1” orthologous genes in five species and used the canonical correlation analysis (CCA) strategy to find linear combinations of features across datasets that were maximally correlated. All five batches of data were finally pooled into a single object. Then, we used unsupervised clustering in integrated ileum datasets. Based on the expression of reported markers (Grun et al., 2015; Haber et al., 2017; Wang et al., 2020b), nine cell types were identified, including enterocytes (*VIL1*, *FABP2*), transient-amplifying (TA) cells (*MKI67*, *STMN1*), goblet cells (*CLCA1*, *AGR2*), goblet progenitor cells (co-expression of goblet and TA cell markers), stem cells (*LGR5*, *SMOC2*), enteroendorcine cells (EECs) (*CHGA*, *CHGB*), Paneth cells (*LYZ*, *MMP7*) and tuft cells (*DCLK1*, *POU2F3*) (Fig. 1C and D). Most of cell types were also found in human, macaque, pig, rat and mouse ileum based on the expression of similar marker genes when separately analyzed (Fig. S1 and Table S2). Interestingly, Paneth cells were absent in rat, pig and macaque ileum, while a new cell population marked by *CA7* (Carbonic anhydrase 7) and *OTOP2* was identified (Fig. 1E). CA7^+^ cells were rarely detected in mouse and rat ileum compared to other species. Tuft cells were not found in human ileum, as reported by the previous report (Wang et al., 2020b).

### Conserved cell types and functions across species

**Fig. 2:**
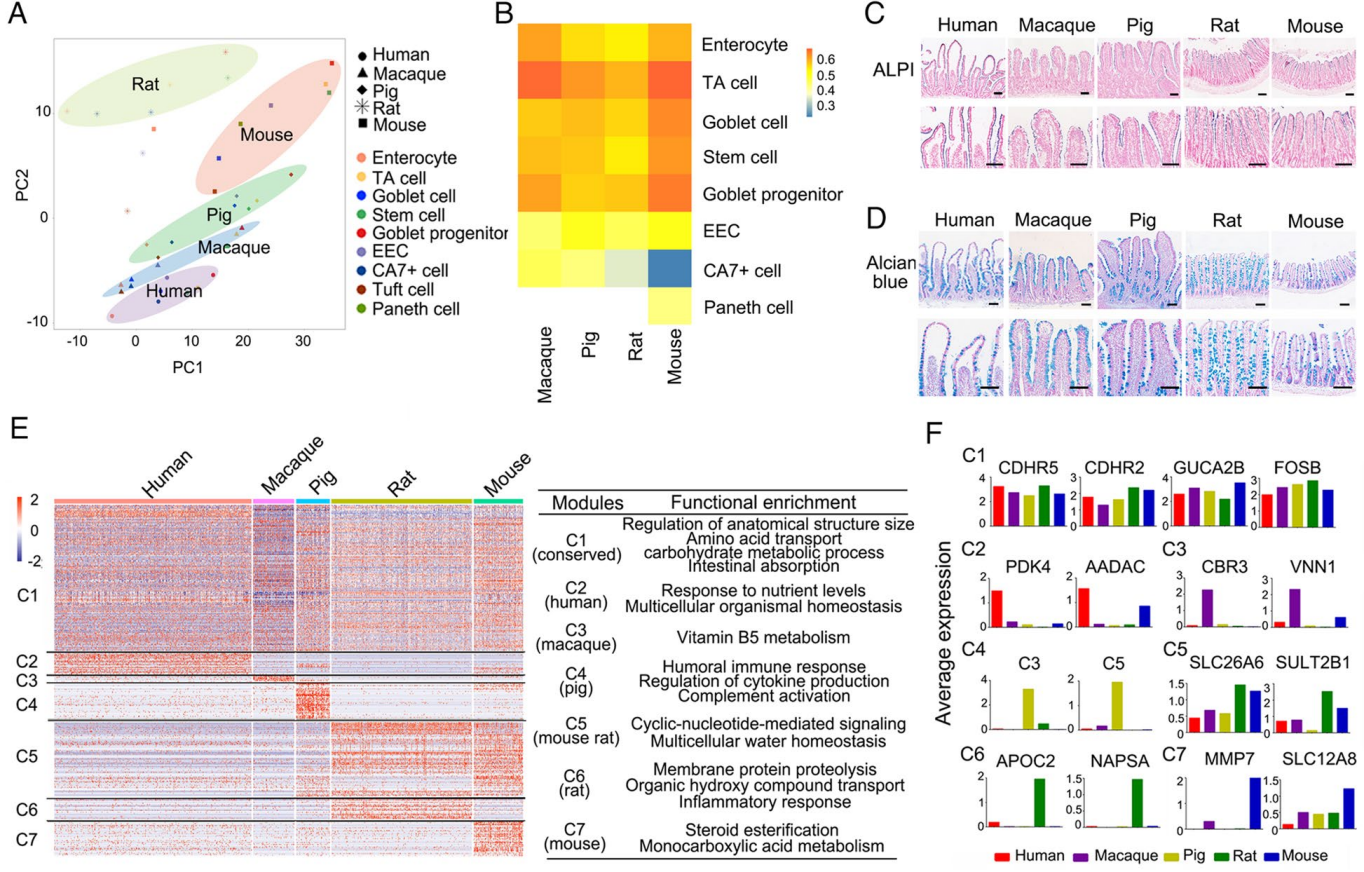
Conserved cell types and functions of the ileum epithelium across species. **A,** PCA plot of all cell types for each species (marked by different shapes). Each dot represents a specific cell type from each species. **B,** Pearson correlation analysis of all cell types of macaque, pig, rat and mouse compared to human. **C, D,** Alpi staining (**C**) and Alcian blue staining (**D**) showing conserved enterocytes and goblet cells across all species. The lower panels show enlargements of the upper panels. Scale bars, 100 μm. **E,** Expression heatmap of conserved and differential genes and functional enrichments across all species. Genes are divided into 7 modules based on the gene expression pattern. **F,** Bar plots of scRNA-seq expression (log1p (average expression)) of signature genes in each module as shown in (**E**)

The ileal epithelium shares the evolutionarily conserved anatomic features and functions in vertebrates (Lickwar et al., 2017), but the environmental pressure and dietary habit influence the intestinal functions in different species (Furness et al., 2015). To explore the cross-species similarity in cell types, principal component analysis (PCA) and correlation analysis of all cell types were performed. PCA showed tight clustering of cell types in each species, except for rat (Fig. 2A), indicating that the inter-species difference was larger than intra-species. Macaque was the closest to human, and rodents (mouse and rat) were far away from human, reflecting the evolutionary relationship. Correlation analysis of each cell type revealed that enterocytes, TA cells, goblet cells, stem cells and goblet progenitor were highly conserved between human and other species (0.53 < R < 0.68), while EECs, CA7^+^ cells and Paneth cells showed more divergent in gene expression across five species (R < 0.50) (Fig. 2B). To further confirm the conservation of cell types, immunochemistry and immunofluorescence were performed. Alpi staining showed the conservation of enterocytes across five species, located in the surface of villus (Fig. 2C). Alcian blue and Muc2 staining identified the existence of goblet cells (Fig. 2D and Fig. S2A). EECs exhibited the scattered distribution in the ileal epithelium of five species shown by ChgA staining (Fig. S2B). TA cells, marked by Ki67, were found in the crypt region in five species (Fig. S2C). In accordance with the scRNA-seq data, Paneth cells were only observed in mouse and human ileum, but not in rat, pig and macaque, as shown by Lyz staining (Fig. S2D).

We then explored the gene function conservatism across species. We compared all cells from each species and examined their gene expression. Based on the gene expression pattern, seven modules were determined, including the conserved module widely expressed in all species (C1) and species-specific modules (C2-C7) (Fig. 2E and Table S3). Functional enrichment analysis revealed that the genes involved in regulation of anatomical structure size, amino acid transport, carbohydrate metabolic process and intestinal absorption were conserved in all species (C1). In addition, each species had the unique functions. For example, several functions including humoral immune response, cytokine production and complement activation were found in pig ileum (C4). The genes involved in membrane protein proteolysis and organic hydroxyl compound transport were highly expressed in rat ileum (C6). The function of steroid esterification was enriched in mouse ileum (C7). The stronger immune response in pig ileum was supported by previous observations that gut microbiota in pig was more abundant compared to human, mouse and rat (Kobayashi et al., 2020; Xiang et al., 2020).

Then, the signature genes in each module were selected as molecular markers for each species (Fig. 2F). Two brush border-specific protocadherins *CDHR2* and *CDHR5* were ubiquitously expressed in all species, which were essential for villus formation and enterocyte function (Crawley et al., 2014; Pinette et al., 2019). *GUCA2B*, which was also broadly expressed across species in C1 module, contributed to ion and fluid transport in gastrointestinal tract as an endogenous activator of intestinal guanylate cyclase (Brenna et al., 2016; Field, 2003). In C2 module, *PDK4* (Pyruvate dehydrogenase kinase 4) was highly expressed in human, which played important roles in glucose and fatty acid metabolism (Zhao et al., 2020). Epithelial pantetheinase *VNN1*, involved in vitamin B5 metabolism (Dupre et al., 1970), was found in macaque (C3). Genes *C3* and *C5*, as components of complement system, were specifically found in pig ileum (C4). Anion transporter *SLC26A6*, mediating chloride absorption and bicarbonate secretion in the small intestine (Seidler et al., 2008; Wang et al., 2020a), was expressed in mouse and rat ileum (C5). The aspartic protease *NAPSA* was enriched in rat ileum, consistent with protein proteolysis in functional enrichment analysis (C6). *MMP7* (Metalloproteinases), a marker for Paneth cells and essential for α-defensins maturation and mucosal protection in mouse intestine (Mastroianni et al., 2012; Vandenbroucke et al., 2014), was enriched in mice (C7). These data indicate that the ileum harbors species-specific gene expression across mammalian species.

### Distinct expression patterns of EECs and Paneth cells among species

**Fig. 3:**
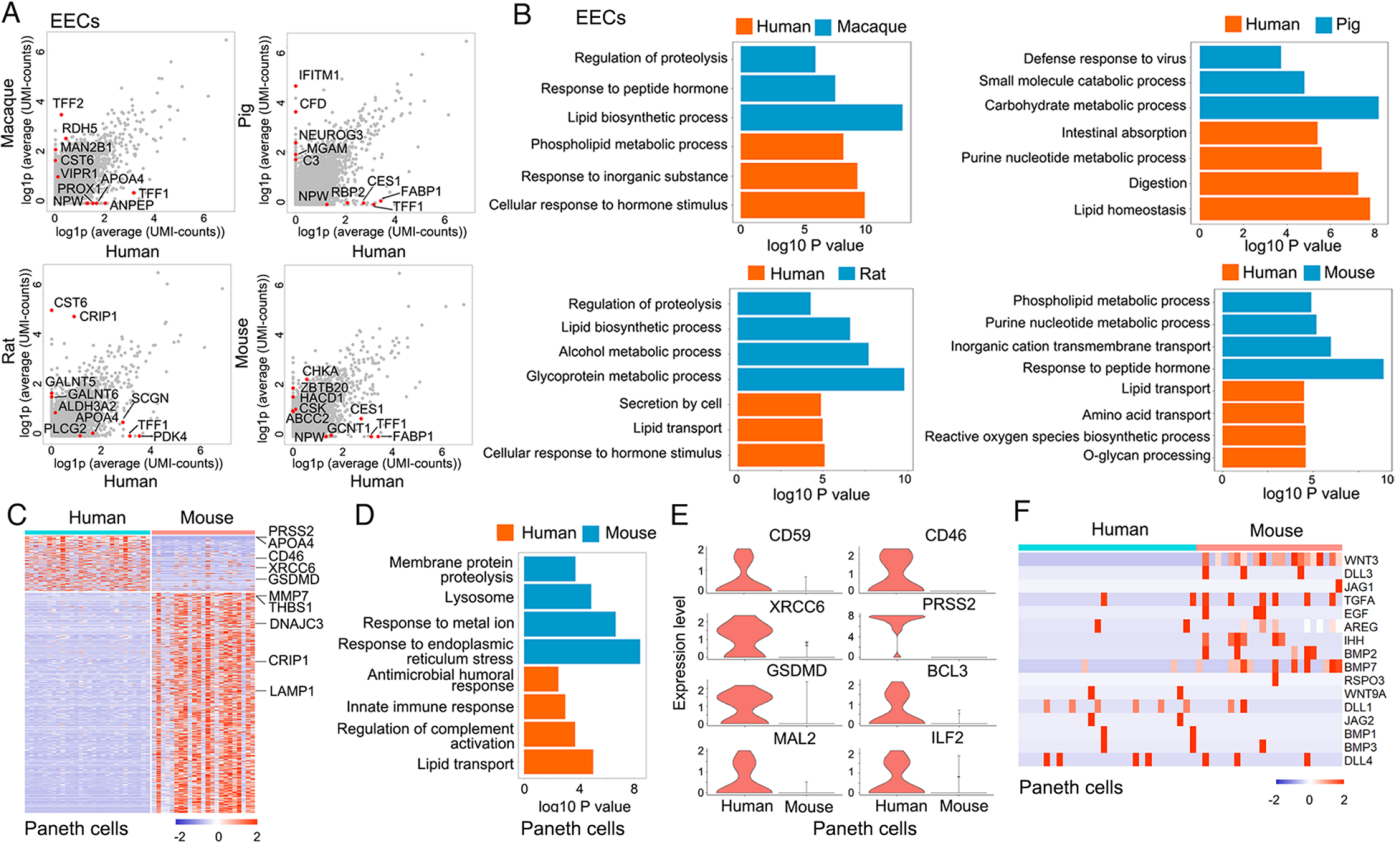
Differential gene expressions of EECs and Paneth cells cross species**. A,** Scatterplot showing differentially expressed genes in EECs between human and other species. Several differentially expressed genes are highlighted in red. **B,** Comparison of functional enrichment of EECs between human and other species. **C,** Expression heatmap of signature genes in human and mouse Paneth cells. **D,** Functional enrichment of Paneth cells in human and mouse. **E,** Violin plots showing expression distributions of immunity-related genes in human and mouse Paneth cells. **F,** Expression heatmap of growth factors in human and mouse Paneth cells

EECs are hormone-producing cells in the intestine, which sense the nutrients and play key roles in regulating the appetite, food digestion and absorption (Fothergill and Furness, 2018; Gribble and Reimann, 2016). Pairwise comparison of human and other species showed a large number of differential expressed genes in EECs (Fig. 3A and Table S4). For instance, ANPEP (Aminopeptidase N), which is involved in the processing of somatostatin and kallidin (Danziger, 2008), was higher in the human ileum compared to macaque. NPW, a neuropeptide regulating the food intake (Li et al., 2018a), was enriched in human compared to other species, except rat. The proteolysis associated gene *CST6* was found in macaque and rat ileum. *GALNT5* and *GALNT6*, involved in mucin O-linked glycosylation process (Detarya et al., 2020; Lavrsen et al., 2018), were specially expressed in rat, consistent with the higher glycoprotein metabolic ability (Fig. 3B). In contrast, MGAM (maltase-glucoamylase) which catalyzes the digestion of starch (Diaz-Sotomayor et al., 2013; Karasov and Caviedes-Vidal, 2021), was highly expressed in pig. Functional enrichment analysis on the differential genes of EECs also showed the similar results (Fig. 3B). In generally, the genes involved in proteolysis and peptide hormone response were expressed in macaque EECs, and the genes related to carbohydrate metabolism and virus defense were enriched in pig.

Paneth cells were identified based on *LYZ* (Lyz1 in mouse) and *DEFA5* expression. Surprisingly, Paneth cells were found only in human and mouse ileum, but not in macaque, pig and rat. *MMP7* is a marker for murine Paneth cells, but was rarely detected in human Paneth cells (Fig. 3C and Table S5). PRSS2, a trypsinogen, was enriched in human Paneth cells (Fig. 3C). The different expression pattern suggested Paneth cells may perform special functions in mouse and human, respectively. Further, functional enrichment analysis showed that the genes involved in lipid transport, complement activation, and innate immune response were expressed in human Paneth cells (Fig. 3D), indicating that human Paneth cells may preferably perform the anti-microbial function. The enriched expression of immunity-related genes in human was consistent with the function (Fig. 3E). In contrast, the genes associated with endoplasmic reticulum stress, response to metal ion, lysosome and membrane protein proteolysis were found in mouse Paneth cells (Fig. 3D).

Paneth cells have been shown to provide niche factors for Lgr5^+^ intestinal stem cells (Sato et al., 2011; Wang et al., 2020b). Examination of the expression of various signal ligands showed that Wnt3 was enriched in murine Paneth cells and rarely detected in human cells (Fig. 3F), consistent with previous report (Busslinger et al., 2021). Similarly, *TGFA* was also highly expressed in murine Paneth cells and minimally expressed in human Paneth cells. *EGF, JAG1*, *DLL3*, *AREG*, *IHH*, *BMP2* and *BMP7* were expressed in murine Paneth cells, while *DLL1*, *JAG2*, *BMP1* and *BMP3* were found in human cells (Fig. 3F). In contrast, the expression of *DLL4* was similar between murine and human Paneth cells.

### Conserved and species-specific stem-cell signature genes

**Fig. 4:**
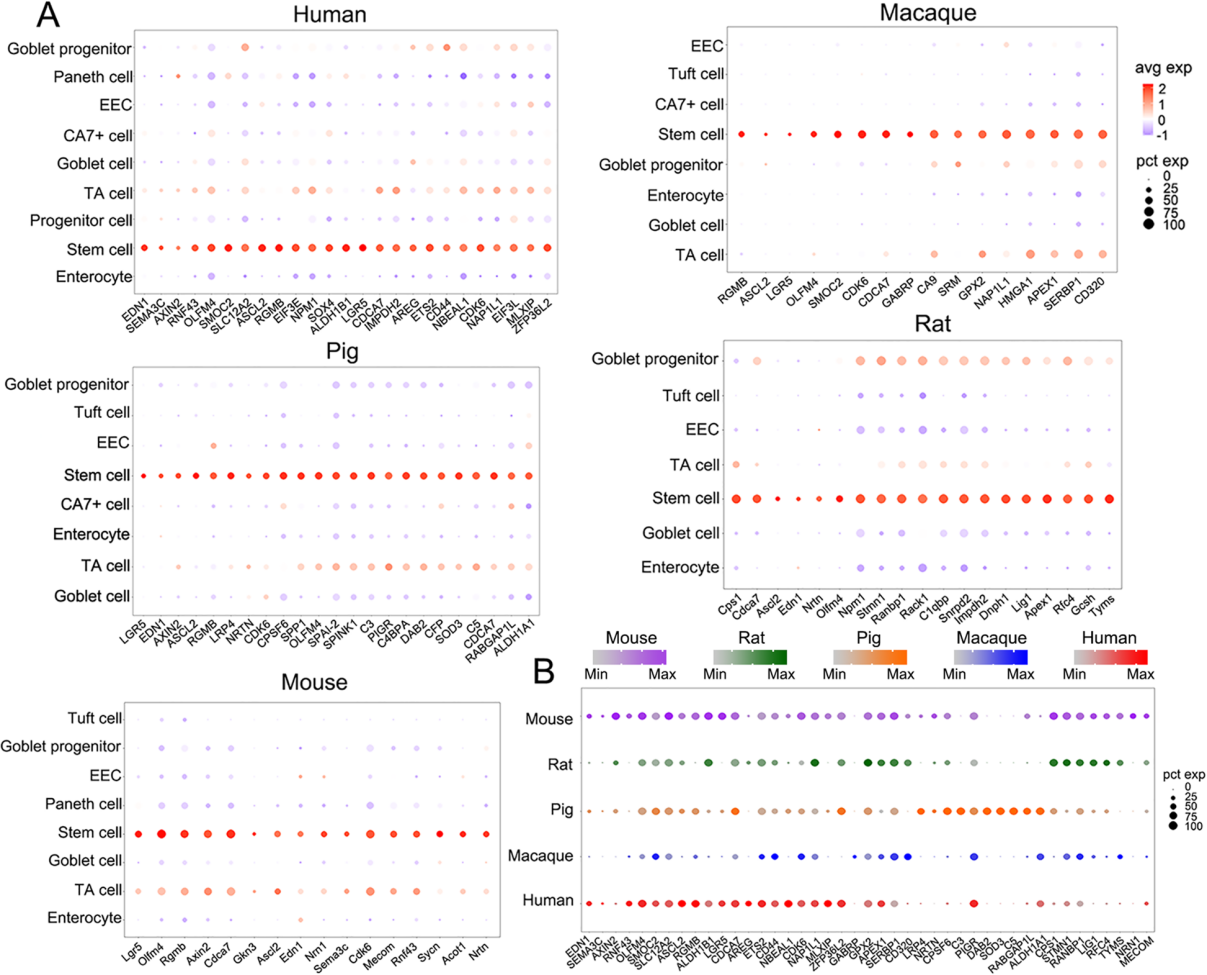
Conserved and species-specific ISC signature genes across species. **A,** Dot plot showing ISC signature genes from human, macaque, pig, rat and mouse ileum. **B,** The expression pattern of ISC signature genes in stem cells from five species. The color indicates the average gene expression, and the size shows the percentage of cells expressing this gene

Intestinal stem cells (ISCs) play a central role in maintaining the intestinal homeostasis and regeneration. Multiple ISC marker genes are reported, including *Lgr5*, *Ascl2*, *Olfm4*, *Smoc2* and *Sox9*. Meanwhile, a comprehensive ISCs signature genes (384 genes) in mouse small intestine have been documented (Munoz et al., 2012). However, ISCs signature genes in other mammals are less understood. Here, we defined ISC signature genes from human (26 genes), macaque (16 genes), pig (23 genes), rat (19 genes) and mouse (16 genes) ileum (Fig. 4A). Among them, 12 were conserved in five species: *OLFM4*, *CDCA7*, *MECOM*, *CDK6*, *RGMB*, *RNF43*, *ASCL2*, *LGR5*, *EDN1*, *SEMA3C*, *AXIN2* and *NRTN*. *OLFM4*, *ASCL2*, *LGR5*, *AXIN2* and *RNF43* are widely recognized as ISC signatures. *CDCA7*, *MECOM* and *CDK6* are involved in the maintenance of hematopoietic stem cells (Guiu et al., 2014; Maicas et al., 2017; Scheicher et al., 2015). *RGMB*, *EDN1*, *SEMA3C* and *NRTN* have been indicated to regulate olfactory neurogenesis, mesenchymal stem cells regeneration, tumorigenicity of glioma stem cells and neurite outgrowth, respectively (Hwang et al., 2021; Kam et al., 2016; Man et al., 2014; Reyes-Corona et al., 2017). Furthermore, several species-specific ISCs signature genes were distinguished. *LRP4* and *SOD3* were highly expressed in stem cells in pig ileum. *NRN1* and *GABRP* were mouse-specific and macaque-specific stem cell signatures, respectively (Fig. 4B). Interestingly, the widely recognized ISC signature *LGR5* was barely detected in rat and macaque, consistent with early reports of its low expression in rat intestine (Dudhwala et al., 2020; Femia et al., 2013). Together, these data provide the valuable information to identify intestinal stem cells in different species.

### Molecular characterization of CA7^+^ cells across species

**Fig. 5:**
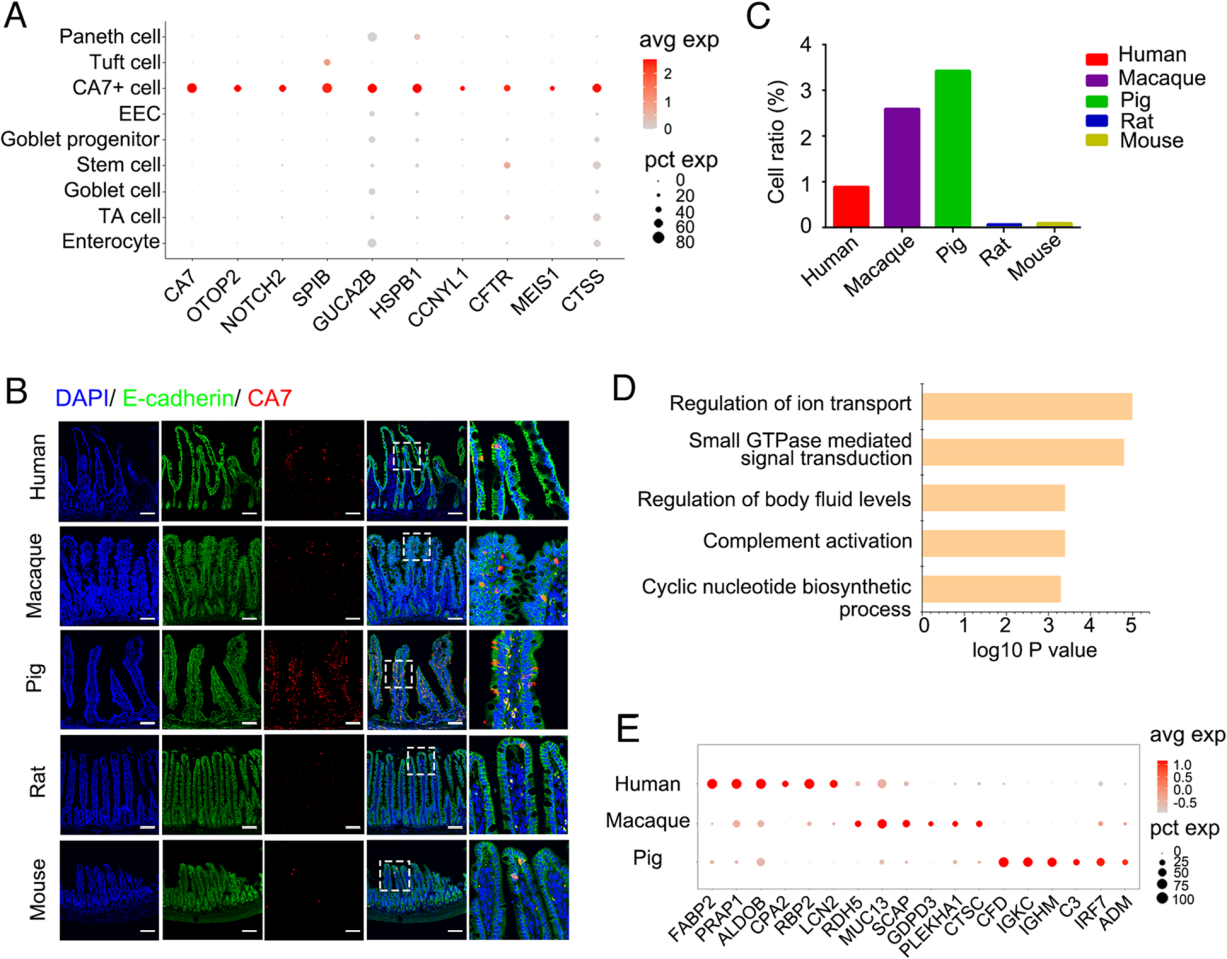
Identification of CA7^+^ cells in pig, macaque and human ileum. **A,** Dot Plot showing signature genes in CA7^+^ cells, colored by scaled expression level. Each dot represents a gene and the size shows the percentage of cells expressing this gene. **B,** Immunofluorescence was performed to confirm the CA7 expression in mouse, rat, pig, macaque and human ileum. Scale bars, 100 μm. **C,** The percentage of CA7^+^ cells in mouse, rat, pig, macaque and human ileum. **D,** Gene ontology from signature genes in CA7^+^ cells. **E,** Dot Plot showing scaled expression level (color scale) and percentage of expressing cells (point size) of the differential signature genes in CA7^+^ cells of human, macaque and pig ileum

Our clustering analysis revealed a new cell type which highly expressed *CA7*, *OTOP2*, *NOTCH2*, *SPIB* and *GUCA2B* (Fig. 5A). We referred this cluster as CA7^+^ cells. CA7 is a member of carbonic anhydrases, which catalyzes the reversible hydration of carbon dioxide and play key roles in luminal acid sensing in the duodenal epithelia (Sjoblom, 2011). OTOP2 encodes a proton-selective channel in various epithelia (Tu et al., 2018). Transcriptional factor SPIB is essential for microfold cell differentiation in the intestine (Kanaya et al., 2012). GUCA2B, encoding satiety peptide uroguanylin, is involved in intestinal fluid and electrolyte transport (Brenna et al., 2016; Rahbi et al., 2012). The expression of *GUCA2B* was enriched in CA7^+^ cells in pig, macaque and human ileum, while broadly expressed in multiple cell types in mouse and rat ileum, including enterocytes, goblet cells and EECs (Fig. S3A). Immunofluorescence confirmed CA7^+^ cells in the villus region (Fig. 5B). The percentage of CA7^+^ cells varied among species and was about 1–3% in human, macaque and pig ileum, but much less in mouse and rat (Fig. 5C).

Gene ontology enrichment analysis highlighted the functions of CA7^+^ cells in regulation of ion transport, body fluid levels, complement activation and small GTPase mediated signal transduction (Fig. 5D). Furthermore, we identified the species-specific signatures of CA7^+^ cells in human, macaque and pig (Fig. 5E, S3B and Table S6). For instance, *ALDOB* participating in glucose metabolic process and *CPA2* (carboxypeptidase) were enriched in human, and steroid metabolism-related genes *RDH5* and *SCAP* were upregulated in macaque, while innate immune response-related genes *CFD*, *IGKC* and *IGHM* were specifically expressed in pig. Gene functional enrichment analysis also revealed that CA7^+^ cells exhibited functional difference in human, macaque and pig ileum (Fig. S3C). The physiological function of CA7^+^ cells in the ileum needs further investigation.

### Divergence of drug absorption across species

**Fig. 6:**
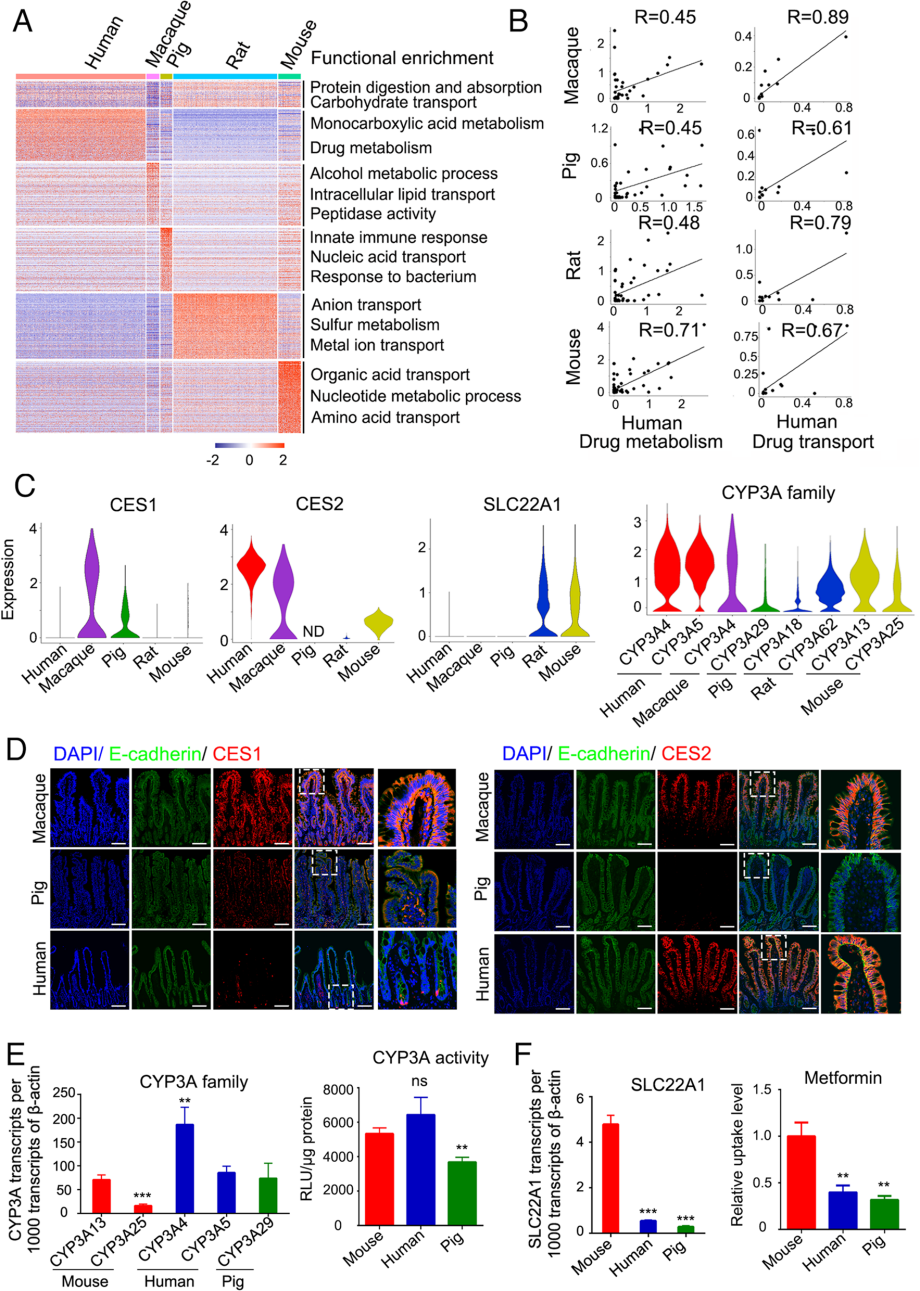
Divergence of drug absorption in ileum. **A,** Expression heatmap of signature genes in enterocytes and functional enrichments among five species. **B,** Pearson correlation of scRNA-seq expression (log1p (average expression)) for drug metabolism and transporter genes. Species from top to bottom: macaque versus human, pig versus human, rat versus human and mouse versus human. **C,** Violin plots showing the expression of drug metabolism and drug transporter genes across human, macaque, pig, rat and mouse. ND, not detected. **D,** Immunofluorescence was performed to confirm the CES1 and CES2 expression in macaque, pig and human ileum. Scale bars, 100 μm. **E,** The expression of CYP3A family members in differentiated mouse, pig and human ileum organoids relative to β-actin, and its activity was examined by P450-Glo CYP3A4 assay kit. **F** *SLC22A1* expression relative to β-actin and metformin uptake in mouse, pig and human ileum organoids. Data are displayed as the mean ± SD (*n* = 3 independent experiments). ns (no significance); *P < 0.05, **P < 0.01, ***P < 0.001 by Student’s t-test (**E** and **F**)

Enterocytes are the major cell type responsible for pharmacokinetic function as they abundantly express transporters and metabolic enzymes (Yoshida et al., 2021). The genes involved in protein digestion and absorption and carbohydrate transport were expressed in all five species (Fig. 6A and Table S7). The genes participating in monocarboxylic acid metabolic process and drug metabolism were highly expressed in human, the genes related to alcohol metabolic process and lipid transport were enriched in macaque, and the genes associated with nucleic acid transport, metal ion transport and organic acid transport and were highly expressed in pig, rat and mouse respectively.

To elucidate the similarity between human and other species in pharmacokinetics, we divided pharmacokinetics-related genes into drug metabolism and drug transporter genes, and calculated Pearson correlation coefficients between human and other species (Fig. 6B, Table S8). The expression of drug metabolism genes in mouse ileum showed the highest correlation with that in human (R = 0.71), whereas the ones in other species showed lower correlation (R = 0.45–0.48). However, the expression of drug transporter genes in macaque ileum showed the highest correlation with that in human (R = 0.89). This is consistent with the report that monkey and human exhibited excellent correlation in oral absorption of 43 drugs (Cheng et al., 2008; Chiou and Buehler, 2002).

Then, we explored several differential genes related to drug metabolism and transport in more details. CES1 and CES2 are two major carboxylesterases which catalyze the hydrolysis of various drugs, including clopidogrel, fenofibrate and irinotecan (Imai et al., 2006; Xu et al., 2002). We found that *CES1* was enriched in macaque ileum, and *CES2* mainly found in human, macaque and mouse ileum (Fig. 6C). The differential expressions of CES1 and CES2 were also confirmed by immunofluorescence (Fig. 6D). CYP3A (cytochrome P450 3A) family members, which are the most abundant CYP enzymes that account for 50% of the metabolism of commonly used drugs in human intestine (Komura and Iwaki, 2008), was highly expressed in human, moderately in macaque, mouse and rat in ileum tissues and organoids (Fig. 6C and E).

To confirm the divergence of drug absorption, we separately established the ileum organoids from mouse, pig and human and induced their differentiation as functional organoids to evaluate the drug metabolism and transport. It showed that mouse is closer to human in term of CYP3A activity (Fig. 6E), supporting the high correlation between mouse and human in drug metabolism. Metformin for the frontline therapy for type II diabetes mellitus is mainly absorbed from the small intestine (Graham et al., 2011; Markowicz-Piasecka et al., 2017), and SLC22A1 is the major transporter for its uptake (Han et al., 2015). The uptake level of metformin was the highest in mouse organoids, compared to human and pig organoids (Fig. 6F), which was also consistent with differential expression of *SLC22A1* in ileum tissues and organoids (Fig. 6C and F).

## Discussion

In this study, we obtained the cross-species ileum transcriptional atlas comprising mouse, rat, pig, macaque and human for the first time and uncovered the conserved and differential cell types and functions across five species. We identified unreported CA7^+^ cells in pig, macaque and human ileum and distinguished the distinct gene expressions of EECs and Paneth cells. In addition, conserved and species-specific ISCs signature genes were defined across five species. Importantly, we elucidated the difference on drug transport and metabolism among five species and established the ileum organoid models for drug absorption studies.

CA7^+^ cells are not defined in the intestinal epithelium. The expression of signature genes (*CA7*, *OTOP2*, *CFTR* and *GUCA2B*) of CA7^+^ cells was similar with BEST4^+^ cells in the human colon (Elmentaite et al., 2021; Parikh et al., 2019) and BCHE cells in the human duodenum (Busslinger et al., 2021), indicating the broad existence of CA7^+^ cells in the human intestine. Carbonic anhydrases catalyze the reversible hydration of CO_2_ and play critical roles in respiration, pH homeostasis, gluconeogenesis, electrolyte secretion and tumorigenicity (Sjoblom, 2011; Supuran, 2008). CA7 as a cytosolic isoform of CAs with high CO_2_ hydration activity, was reported to participate in antioxidant defense processes (Monti et al., 2017). The expression of CA7 was downregulated in colorectal cancer and correlated with disease progression (Yang et al., 2015). Proton-selective channel OTOP2 also acts a tumor suppressor gene in colorectal cancer (Qu et al., 2019). CFTR channel is essential for high-volume fluid secretion of water, chloride and bicarbonate in the intestine (Moran, 2017). Uroguanylin (encoded by *GUCA2B*) could bind to the guanylate cyclase-C receptor and activate the CFTR to regulate the fluid secretion and exhibit the downregulated expression in inflammatory bowel disease (Brenna et al., 2015; Field, 2003). These reports are consistent with our observation of the potential role of CA7^+^ cells in ion transport and body fluid regulation in the intestine shown by enrichment analysis. The cellular origin of the satiety peptide uroguanylin (encoded by GUCA2B) is debated for a long time and different among species (Brenna et al., 2016; Cui et al., 2000; Kokrashvili et al., 2009). We found that the expression of *GUCA2B* was enriched in CA7^+^ cells in pig, macaque and human ileum. Further functional validation of CA7^+^ cells is needed to understand their importance in the intestine.

Interestingly, the well described Paneth cells in mouse and human ileum were not observed in rat, pig and macaque ileum. One possibility is the lower cell number of Paneth cells in intestinal epithelial cells from these species. Previous scRNA-seq surveys showed that the percentage of Paneth cells was dramatically decreased to 0.12% of all ileum epithelial cells in neonatal piglets (21 days) (Meng et al., 2021). In another study comprising the ileum single-cell data from Rhesus monkey, Paneth cells were not detected in ileum epithelial cells (Ziegler et al., 2020). It is possible that other cell types may partially substitute for Paneth cells to perform the anti-microbiota function in rat, pig and macaque ileum. Interestingly, we found that mouse Paneth cells could secrete Wnt ligands (WNT3 and RSPO3) to support ISCs, whereas human Paneth cells not. The sources of Wnt signaling in human intestine may come from other types of cells, such as telocytes, PDGFR-α^+^ pericryptal stromal cells and Gli1^+^ mesenchymal cells (Degirmenci et al., 2018; Greicius et al., 2018; Zhu et al., 2021).

## Conclusions

Our data revealed the differential gene expressions involved in drug transport and metabolism across five species, suggesting that mouse is more similar with human in drug metabolism and macaque is closer to human in drug transport. It is consistent with previous reports showing oral absorption in macaque were similar to human by 103 drugs evaluation (Ward and Smith, 2004). The low correlation between human and macaque in drug metabolism may due to the significant lower drug bioavailability in macaque compared to human (Takahashi et al., 2008; Takahashi et al., 2009). Meanwhile, our data also showed that rat and human shared the similar gene expression patterns in drug transport, in line with the previous report (Cao et al., 2006). Although further experimental data are needed in future, our findings provide a foundation for better understanding of cell constitution and function across species as well as animal model selection and drug evaluation.

## Methods

### Animals

Mice and rats were maintained in the Animal Facility of Tsinghua University. The experiments were approved by IACUC (Institutional Animal Care and Use Committee) of Tsinghua University (YGC-19). Bama miniature pigs were used in this study from Beijing Farm Animal Research Center (affiliated to Institute of Zoology, Chinese Academy of Sciences). The experimental protocols were approved by the Animal Ethics Committee of the Institute of Zoology, Chinese Academy of Sciences (IOZ20180061). The tissues of cynomolgus monkey (*Macaca fascicularis*) were acquired from Yunnan Key Laboratory of Primate Biomedical Research, and the experiments involving cynomolgus monkey were approved by IACUC of Yunnan Key Laboratory of Primate Biomedical Research (LPBR202004012).

### Human ileum tissue collection and ethics statement

The ileum mucosa was freshly acquired at least 10 cm away from the tumor border in surgically resected specimens at Peking University Third Hospital, reported before (Wang et al., 2020b). All samples were obtained with informed consent, and this study was approved by the Peking University Third Hospital Medical Science Research Ethics Committee (M2018083). The relevant ethical regulations were followed by Peking University Third Hospital Medical Science Research Ethics Committee.

### Ileum samples preparation for scRNA-seq

The ileum tissue was extracted from three adult mice (10 weeks), three adult rats (3 months old), three adult pigs (6 months old) and two cynomolgus monkeys (14 years old). A small fragment in the ileum region about 5 cm prior to the cecum was isolated from different species. The ileum epithelial cells were acquired as previously described (Li et al., 2021; Wang et al., 2020b). The digestion protocol was varied in species. The single-cell suspension of mouse ileum was acquired by Tryple (Invitrogen) digestion for 15 min at 37 °C. For rat, pig and macaque ileum, the epithelial cells were incubated with 2 mg/ml collagenase I (Sigma-Aldrich) in Advanced DMEM F12 for 15 min at 37 °C. Then, the sediment was transferred into Tryple for 10 min at 37 °C. Propidium iodide (PI; 5 μg/ml) was used to stain the cell suspension and PI-negative cells were sorted by FACS (Beckman). Single cells were loaded onto the single cell chip from 10X genomics Chromium Single Cell 3′ Solution. The cDNA library was constructed according to instruction and sequenced by Illumina Novaseq 6000 sequencer (Illumina, San Diego, CA, USA) with paired-end 150-bp reads.

### Ortholog gene selection

To compare transcription between species, we first created a gene ortholog list using the human genes as the reference. We download homologous gene lists from Ensemble BioMart (https://asia.ensembl.org/biomart/martview/efb2456d7ea6a4d37b6a2a9f03499a88). Other four species were compared to human and a high-quality ortholog genes list was extracted. To account for gene paralogs and gene-duplication events, an aggregated table of “meta-genes” was created. Each meta-gene may include all gene symbols homologous to one human gene. For each organism, read counts were combined across all manifestations of each meta-gene. Finally, we sorted out 10,779 orthologous genes across 5 species, including 10,118 “1–1–1-1-1” orthologous genes and 661 “1-many” genes (Table S9).

### scRNA-seq low-level processing and filtering

Raw reads were aligned to the different species genome (Human: GRCh38/hg38, Macaque: Mmul_10, Mouse: GRCm38/mm10, Rat: Rnor_6.0, Pig: Sscrofa11.1), and Cell Ranger (v3.1.0) (Zheng et al., 2017) was used to estimate unique molecular identifiers (UMIs). Raw aligned features were loaded and processed using the Seurat package (v4.0.2) (Hao et al., 2021) in R version 4.0.5. Low-quality cells were filtered if they expressed no more than 200 genes or with more than 20% of mitochondrial genes.

### scRNA-seq normalization and clustering

Data normalization was performed using Seurat “NormalizeData” and using “LogNormalize” as the normalization method (sacle.factor = 100,000). Variable genes were detected using “FindVariableFeatures”. We used “FindIntegrationAnchors” to combine the scRNA-seq libraries of the five species. The five batches of scRNA-seq data from human, macaque, pig, rat and mouse were subjected to batch correction as described previously (Mayer et al., 2018). We used the canonical correlation analysis (CCA) strategy to find linear combinations of features across datasets that were maximally correlated. The shared correlation structure conserved among the five datasets. Based on the shared structure, all five batches of data were finally pooled into a single object for downstream analysis (Butler et al., 2018; Hardoon et al., 2004). The scaled gene expression data were projected onto principal components (PC). The first 30 PC were used for non-linear dimensionality reduction using Uniform Manifold Approximation and Projection (UMAP). Clustering was performed using the “FindNeighbors” followed by the “FindClusters” functions. Marker genes for each cluster have been identified using “FindAllMarkers” function.

### scRNA-seq differential gene expression analysis

To identify signature genes of each cell types, functions “FindAllMarkers” and “FindMarkers” in Seurat were used. The function “FindMarkers” was used for identification of signature genes by comparing the cell type of interest to another specific group of cells. Functional enrichment analysis was performed using the online software Metascape (http://metascape.org/) tool with default parameters. PCA and Pearson’s correlation analysis were performed using R software.

### Scatterplots

To generate scatterplots in Fig. 3A, we used Pearson correlation of log1p (average (UMI-counts)). All genes were plotted unless an orthologous gene did not exist in one of the two compared species.

### Ileum organoid culture from mouse, pig and human

Ileum organoids from human, mouse and pig were described as previously described (Li et al., 2021) (Li et al., 2018b) (Wang et al., 2020b). The ileum tissue was cut longitudinally and washed by cold PBS for 5–6 times to remove the contaminant and feces. Villi were carefully scraped away and the tissue was cut into several small pieces (about 10 cm). Then, small pieces were incubated in 10 mM EDTA in PBS for 30 min on ice and the crypts were acquired by vigorously scrapping. After centrifugation (3 min at 1000 rpm), the crypts were embedded into Matrigel (BD Biosciences) and seeded on 24-well plate. After polymerization, the culture medium was added. Advanced DMEM/F12 was supplemented with 2 mM GlutaMAX, 1 mM N-acetylcysteine, 1X N2, 1X B-27 and penicillin/streptomycin to prepare a basal medium (all from Thermo Fisher). The organoid culture medium for mouse ileum (ENR) included 50 ng/mL EGF (Invitrogen), 100 ng/mL Noggin (R&D Systems), 500 ng/mL R-spondin-1 (R&D Systems) in basal medium. The organoid medium for pig and human ileum was supplemented with 50 ng/mL EGF (Invitrogen), 100 ng/mL Noggin (R&D Systems), 500 ng/mL R-spondin-1 (R&D Systems), 10 mM Nicotinamide (Sigma-Aldrich), 5 μM CHIR-99021 (Selleck), 0.5 μM A-83-01 (Cayman), 10 μM SB202190 (Selleck), 10 μM Y27632 (Enzo) and 2.5 μM PGE2 (Selleck) in basal medium. Growth medium was replaced every 3–4 days.

### Immunofluorescence

Immunofluorescence was performed as previously described (Qi et al., 2017). Briefly, the ileum tissues from mouse, rat, pig, macaque and human were washed in cold PBS and were fixed in 4% paraformaldehyde for overnight at room temperature. Then paraffin-embedded ileum sections were de-paraffinized in isopropanol and dehydrated by a graded alcohol series, followed by antigen retrieval. Next, the sections were washed by PBS for 3 times and permeabilized by 0.1% Triton X-100 for 15 min at room temperature. Then, the sections were blocked with PBT solution (3% BSA and 0.01% Triton X-100 in PBS) for 1 h at room temperature, followed by incubating with primary antibodies overnight at 4 °C. The fluorescein-labeled secondary antibodies (Life Technologies, 1:300) and 4′, 6-diamidino-2-phenylindole (DAPI) were added for 1 h at room temperature next day. The images were acquired from Olympus FV3000 Laser Scanning Microscope.

### Antibodies

Rabbit anti-Muc2 (1:200, sc-15,334; Santa Cruz), rabbit anti-ChgA (1:200, ab15160; Abcam), rabbit anti-Ki67 (1:200, ab15580; Abcam), rabbit anti-Lyz (1:200, ab108508; Abcam), mouse anti-E cadherin (1:200, 610,182; BD Biosciences), rabbit anti-Ca7 (1:200, 13,670–1-AP; Proteintech), rabbit anti-Ces1(1:200, 16,912–1-AP, Proteintech), rabbit anti-Ces2 (1:200, ab184957, Abcam).

### Hematoxylin-eosin (H&E), alkaline phosphatase (ALPI) and Alcian blue staining

The ileum tissue was fixed with 4% formalin overnight and embedded in paraffin. The sections (5 μm) were de-paraffinized in isopropanol and graded alcohols. Then, sections were stained by Hematoxylin-eosin (H&E) kit (Beyotime), Alkaline phosphatase (ALPI) kit (Beyotime) and Alcian blue staining Alcian blue kit (BASO) according to manufacturer’s instructions. For H&E staining, sections were stained with hematoxylin solution for 6 min, followed by differentiated medium in 1% acid alcohol for 2 s. Then, the sections were stained with eosin for 2 min (C0105, Beyotime). For ALPI staining, Alkaline phosphatase solutions were added into the sections for 15 min at room temperature. Then, the sections were stained with nuclear fast red for 1 min (C3206, Beyotime). For Alcian blue staining, the sections were stained with Alcian blue for 15 min and nuclear fast red for 1 min (BA4087B, Baso). The images were obtained with a Nikon microscope.

### RNA extraction and quantitative RT–PCR

The total RNA from organoids was extracted by RNeasy Mini Kit (Qiagen). The cDNA was obtained by Revertra Ace (Toyobo). Then, real-time PCR reactions were performed using qPCR Master Mix (Promega) in triplicates on a LightCycler 480 (Roche). The primers of selected gene were shown in Supplementary Table S10. The experiments were performed with three biological replicates.

### CYP3A activity

To measure the activity of CYP3A4 and its homologues in mouse, pig and human ileum organoids, the non-lytic assays were performed by using a P450-Glo CYP3A4 assay kit (V9001; Promega) according to the manufacturer’s instructions. Briefly, luciferin-IPA (CYP3A4 substrate) in fresh culture medium was added into the organoids for 1 h at 37 °C. Then, the medium was transferred into white luminometer plate and detection reagent was added for 20 min at room temperature. The CYP3A activity was measured through fluorescence signals with luminometer and normalized with the protein content per well by BCA protein kit (P0012S, Beyotime).

### Drug absorption assay

Mouse, pig and human ileum organoids were seeded into 24 well plates in the indicated differentiation media. After 3 days, metformin (obtained from MCE) was separately added into the media for three kinds of differentiated organoids. After 12 h incubation, the organoid cells were isolated to detect the metformin absorption levels. Then, four volumes of acetonitrile with 0.1% methanoic acid were added into cells. The mixture was performed centrifugation (10 min at 12000 rpm, 4 °C) and 200 μl supernatant was extracted to detect the content of drugs by liquid chromatography mass spectrometry (LC-MS/MS; Q Exactive; Thermo Scientific). The drug absorption level was normalized with live cell numbers per well. Organoids were incubated with Tryple (Invitrogen) for 20 min at 37 °C to obtain the single-cell suspension. Acridine Orange and PI were added into the suspension to calculate the live cell numbers by fluorescent cell counter (Luna; Logos Biosystems). The relative uptake level from mouse, pig and human ileum organoids was calculated by comparing with mouse ileum organoids.

### Statistical analysis

All experiments were performed with at least three biological replicates. Data shown in column graphs indicated the mean ± SD. Student’s t-test, one-way ANOVA and two-way ANOVA analysis were used to compare difference between two groups as indicated in the figure legends. *P < 0.05, **P < 0.01, ***P < 0.001. The statistical analysis was carried in GraphPad Prism 6 software. The villus length of ileum was quantified with Image J.

## Supplementary Information

**Additional file 1: Table S1.** Shows an overview of the scRNA-seq database and cell type distribution from each species.

**Additional file 2: Table S2.** Shows genes in in different cell types of macaque, pig, rat and mouse ileum in Fig. S1.

**Additional file 3: Table S3.** Shows conserved and distinct gene modules across five species in Fig. 2E.

**Additional file 4: Table S4.** Shows differentially expressed genes in EECs between human and other species in Fig. 3A.

**Additional file 5: Table S5.** Shows differentially expressed genes in murine and human Paneth cells in Fig. 3C.

**Additional file 6: Table S6.** Shows differentially expressed genes in CA7+ cells in human, macaque and pig in Fig. S3B.

**Additional file 7: Table S7.** Shows conserved and differentially expressed genes in enterocytes across five species in Fig. 6A.

**Additional file 8: Table S8.** Shows correlation score in drug metabolism and transport between human and other species in Fig. 6B.

**Additional file 9: Table S9.** Shows orthologous gene lists in mouse, rat, pig, macaque and human.

**Additional file 10: Table S10.** Shows quantitative PCR primers.

**Additional file 11: Figs. S1.** Cell landscapes of ileum epithelial cells in each species based on single-cell RNA-seq data. A, C, E, G, I, UMAP plots showing different cell types from ileum epithelial cells in human (A), macaque (C), pig (E), rat (G) and mouse (I). B, D, F, H, J, Dot Plot showing cell type-specific genes in human (B), macaque (D), pig (F), rat (H) and mouse (J) ileum. Each dot represents a gene. The color indicates the average gene expression and the size shows the percentage of cells expressing this gene. **Figs. S2.** Cell type identification by marker gene expression across species. A-D Muc2 staining (A), ChgA staining (B), Ki67 staining (C), Lyz staining (D) in ileum sections from mouse, rat, pig, macaque and human to show goblet cells, enteroendocrine cells, TA cells and Paneth cells, respectively. Scale bars, 100 μm. **Figs. S3.** Differential expression patterns and functions in CA7+ cells across species. A, Violin plots showing expression distributions of GUCA2B across species. B, C, Expression heatmap (B) and functional enrichments (C) of signature genes in CA7+ cells of human, macaque and pig ileum. A subset of differentially expressed genes was shown in the heatmap.

## Data Availability

The scRNA-seq data, generated in this study are publicly available through the Gene Expression Omnibus (GEO) with the accession code GSE196663.

## Abbreviations

CCA: canonical correlation analysis
CYP3A: cytochrome P450 3A
EEC: enteroendorcine cell
ISC: intestinal stem cell
PC: principal components
PCA: principal component analysis
scRNA-seq: single cell RNA-sequencing
TA cell: transient-amplifying cell
UMAP: Uniform Manifold Approximation and Projection
UMI: unique molecular identifier
