# Supplementary material for: Cross-species single-cell transcriptomic analysis reveals divergence of cell composition and functions in mammalian ileum epithelium
Source: Cell Regen. 2022 May 5;11:19. doi: 10.1186/s13619-022-00118-7 (PMC9072607; doi:10.1186/s13619-022-00118-7)
Supplement: Supplementary file 11 — Additional file 11: Figs. S1. Cell landscapes of ileum epithelial cells in each species based on single-cell RNA-seq data. A, C, E, G, I, UMAP plots showing different cell types from ileum epithelial cells in human (A), macaque (C), pig (E), rat (G) and mouse (I). B, D, F, H, J, Dot Plot showing cell type-specific genes in human (B), macaque (D), pig (F), rat (H) and mouse (J) ileum. Each dot represents a gene. The color indicates the average gene expression and the size shows the percentage of cells expressing this gene. Figs. S2. Cell type identification by marker gene expression across species. A-D Muc2 staining (A), ChgA staining (B), Ki67 staining (C), Lyz staining (D) in ileum sections from mouse, rat, pig, macaque and human to show goblet cells, enteroendocrine cells, TA cells and Paneth cells, respectively. Scale bars, 100 μm. Figs. S3. Differential expression patterns and functions in CA7+ cells across species. A, Violin plots showing expression distributions of GUCA2B across species. B, C, Expression heatmap (B) and functional enrichments (C) of signature genes in CA7+ cells of human, macaque and pig ileum. A subset of differentially expressed genes was shown in the heatmap. [file 13619_2022_118_MOESM11_ESM.pdf]

Supplementary Materials

Cross-species single-cell transcriptomic analysis reveals divergence of cell composition and functions in mammalian ileum epithelium

Haonan Li[1,*], Xiaodan Wang[1,*], Yalong Wang[1,2], Mengxian Zhang[1], Fan Hong[3], Hong Wang[4], Along Cui[5], Jianguo Zhao[5], Weizhi Ji[4] and Ye-Guang Chen[1,3,#]

Supplemental Figure S1-3

Supplemental Table S1-10

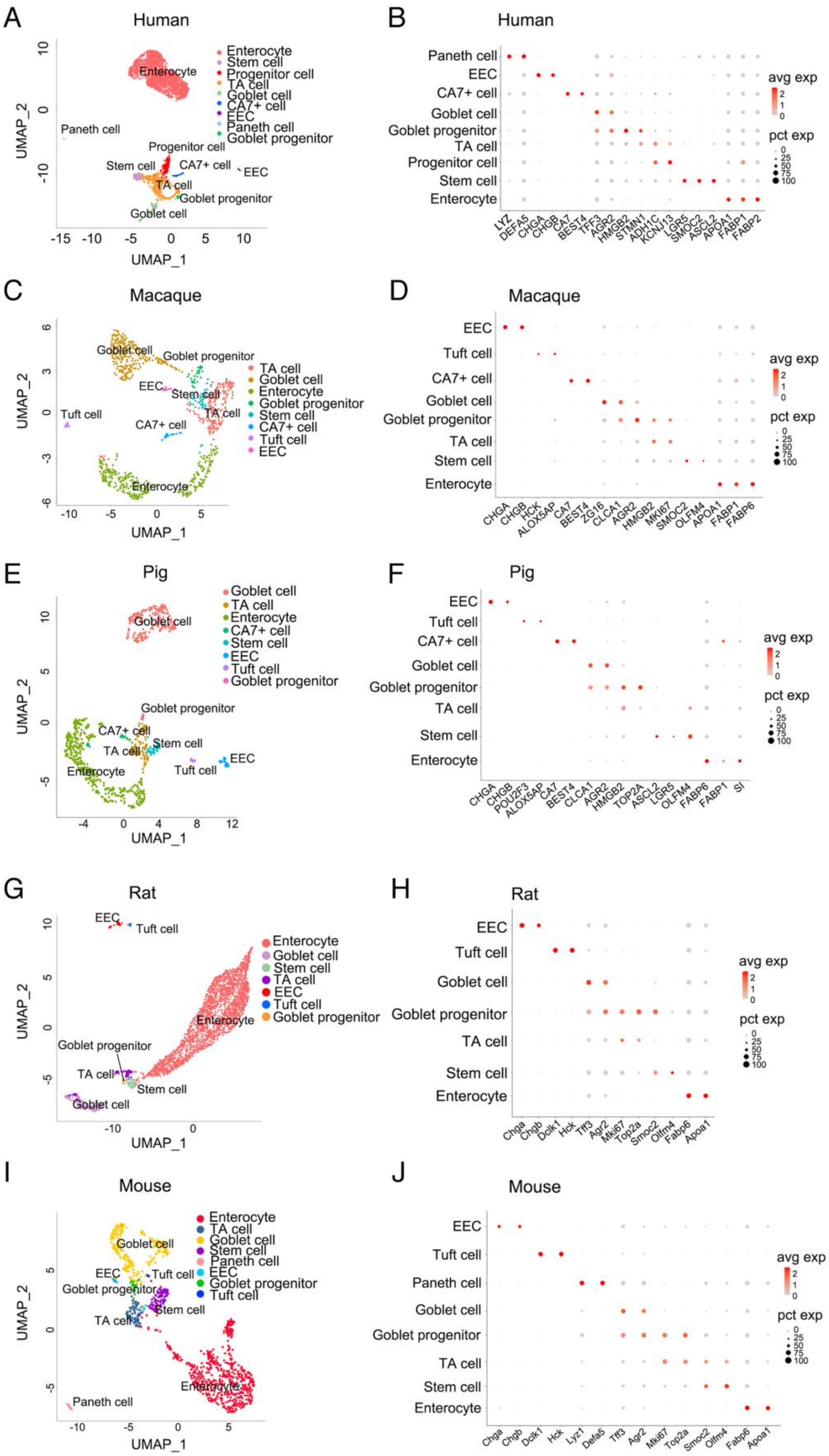
A
Human
Enterocyte
Stem cell
Progenitor cell
TA cell
Goblet cell
CA7+ cell
EEC
Paneth cell
Goblet progenitor
UMAP_1
UMAP_2
B
Human
Paneth cell
EEC
CA7+ cell
Goblet cell
Goblet progenitor
TA cell
Progenitor cell
Stem cell
Enterocyte
avg exp
pct exp
LYZ DEFA5 CHGA CHGB CA7 BEST4 TFF3 AGR2 HMGB2 STMN1 ADH1C KCNJ13 LGR5 SMOC2 ASCL2 APOA1 FABP1 FABP2
C
Macaque
TA cell
Goblet cell
Enterocyte
Goblet progenitor
Stem cell
CA7+ cell
Tuft cell
EEC
D
Macaque
CHGA CHGB HCK ALOX5AP CA7 BEST4 ZG16 CLCA1 AGR2 HMGB2 MKI67 SMOC2 OLFM4 APOA1 FABP1 FABP6
E
Pig
Goblet cell
TA cell
Enterocyte
CA7+ cell
Stem cell
EEC
Tuft cell
Goblet progenitor
F
Pig
CHGA CHGB POU2F3 ALOX5AP CA7 BEST4 CLCA1 AGR2 HMGB2 TOP2A ASCL2 LGR5 OLFM4 FABP6 FABP1 SI
G
Rat
Enterocyte
Goblet cell
Stem cell
TA cell
EEC
Tuft cell
Goblet progenitor
H
Rat
Chga Chgb Dclk1 Hck Tff3 Agr2 Mki67 Top2a Smoc2 Olfm4 Fabp6 Apoa1
I
Mouse
Enterocyte
TA cell
Goblet cell
Stem cell
Paneth cell
EEC
Goblet progenitor
Tuft cell
J
Mouse
Chga Chgb Dclk1 Hck Lyz1 Defa5 Tff3 Agr2 Mki67 Top2a Smoc2 Olfm4 Fabp6 Apoa1

Figures S1 Cell landscapes of ileum epithelial cells in each species based on single-cell RNA-seq data. A, C, E, G, I, UMAP plots showing different cell types from ileum epithelial cells in human (**A**), macaque (**C**), pig (**E**), rat (**G**) and mouse (**I**). **B, D, F, H, J,** Dot Plot showing cell type-specific genes in human (**B**), macaque (**D**), pig (**F**), rat (**H**) and mouse (**J**) ileum. Each dot represents a gene. The color indicates the average gene expression and the size shows the percentage of cells expressing this gene.

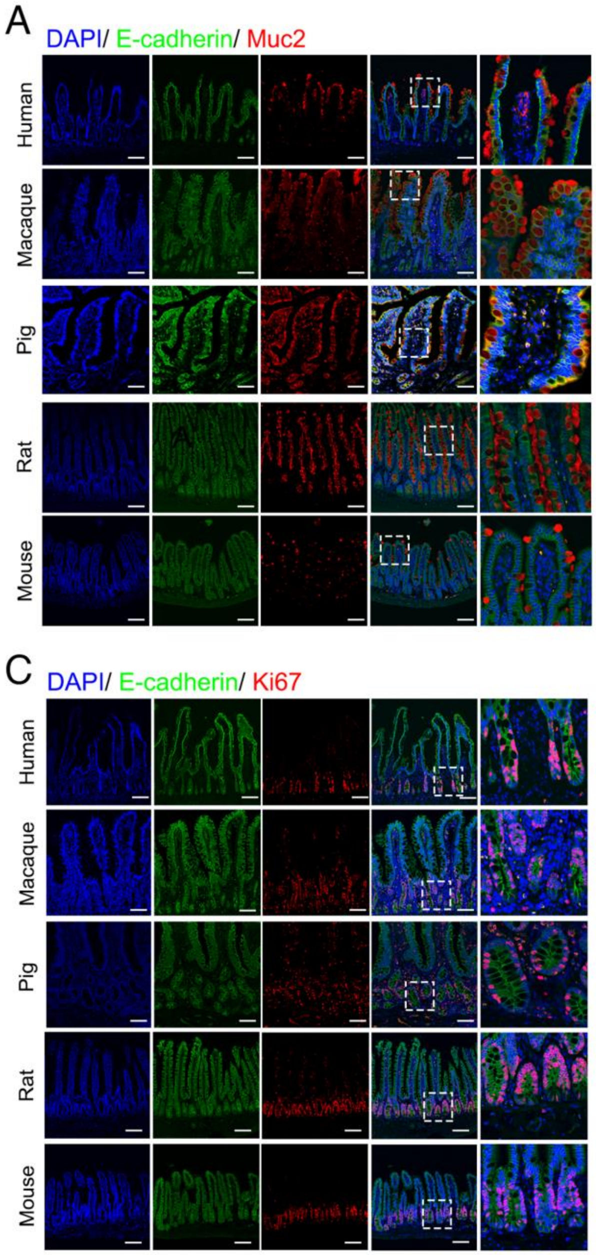

Figures S2 Cell type identification by marker gene expression across species. A-D Muc2 staining (A), ChgA staining (B), Ki67 staining (C), Lyz staining (D) in ileum sections from mouse, rat, pig, macaque and human to show goblet cells, enteroendocrine cells, TA cells and Paneth cells, respectively. Scale bars, 100μm.

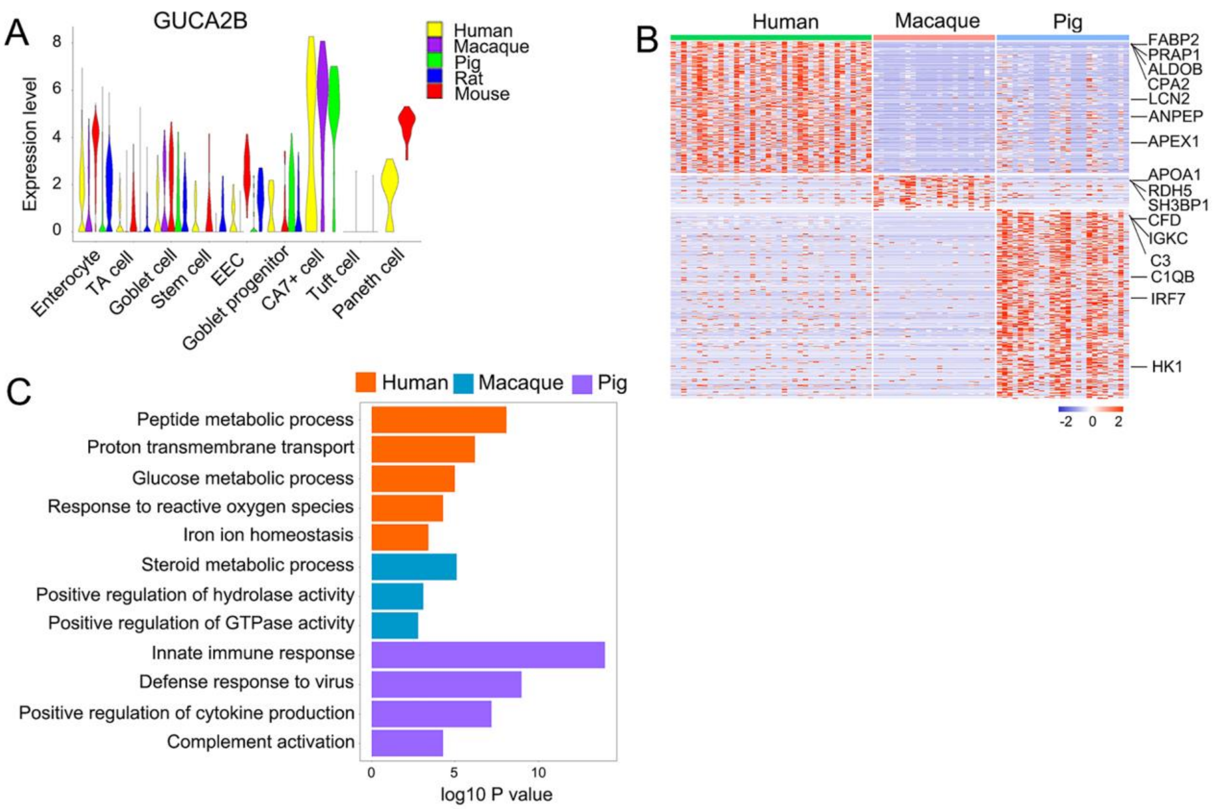

Figures S3 Differential expression patterns and functions in CA7^{+} cells across species. A, Violin plots showing expression distributions of *GUCA2B* across species. **B, C,** Expression heatmap (**B**) and functional enrichments (**C**) of signature genes in CA7^{+} cells of human, macaque and pig ileum. A subset of differentially expressed genes was shown in the heatmap.

Table S1 shows an overview of the scRNA-seq database and cell type distribution from each species.

Table S2 shows genes in in different cell types of macaque, pig, rat and mouse ileum in Fig. S1.

Table S3 shows conserved and distinct gene modules across five species in Fig. 2E.

Table S4 shows differentially expressed genes in EECs between human and other species in Fig. 3A.

Table S5 shows differentially expressed genes in murine and human Paneth cells in Fig. 3C.

Table S6 shows differentially expressed genes in $CA7^{+}$ cells in human, macaque and pig in Fig. S3B.

Table S7 shows conserved and differentially expressed genes in enterocytes across five species in Fig. 6A.

Table S8 shows correlation score in drug metabolism and transport between human and other species in Fig. 6B.

Table S9 shows orthologous gene lists in mouse, rat, pig, macaque and human .

Table S10 shows quantitative PCR primers.
